# Engaging men in HIV services in sub-Saharan Africa: an authors' viewpoint on what has been done and what still needs to be done

**DOI:** 10.11604/pamj.2020.37.58.23062

**Published:** 2020-09-15

**Authors:** Moreblessing Chipo Mashora

**Affiliations:** 1 Department of Public Health, Mount Kenya University, Kigali, Rwanda.

**Keywords:** Men, health services, HIV

## Abstract

There has been an increased recognition of the importance of men in the global HIV response. Previous studies indicate that in different settings, men are generally less engaged in various HIV services besides having worse health outcomes in comparison to the women. Some of the main factors behind this, based on the previous studies include social factors, gender factors, economic factors, political factors, as well as institutional factors. Recently, various scholars have been reporting evidence concerning the strategies, which aims to improve the levels of engagement of men when it comes to HIV services. There are a number of highly promising approaches, which have been suggested, which includes community-based outreach programs, gender-transformative interventions aimed at shifting gender practices and norms, as well as the development of highly responsive and male-friendly health services. Despite the fact that a number of initiatives have been put in place, there are different kind of challenges, which still remain, more so with regards to costs, as well as sustainability, intersecting inequalities such as class and race, and the challenges, which are faced with regards to altering the community-level gender norms.

## Perspective

**The case to engage men in HIV services:** men are affected by the HIV epidemic because of the harmful gender norms as well as inadequate health systems for responding to the different needs of men. Men are generally less likely to access HIV testing, and besides, they are always less likely to seek, use and adhere to the antiretroviral therapy. Additionally, they generally tend to have a lower CD4 count at the initiation of treatment. Men are also highly likely to die while on antiretroviral therapy [1]. While this is the case, the strategies, which have been used to ensure that men are highly engaged in HIV services in sub-Saharan Africa, have not been highly effective in generating the desired outcomes.

**Interventions adopted to engage men in HIV services. What has worked?:** the past half-decade has seen various discussions concerning the need to ensure active engagement of the men in sub-Saharan Africa with regards to HIV prevention campaigns [2]. Sharma [1] indicated that some of the notable interventions, which have been used include active partner notification services, which provided exposure notification as well as community based HIV testing and counseling HTC for the sexual partners of the newly diagnosed HIV-positive individuals. The other notable interventions based on the study include the use of mobile HIV testing as well as counseling. This is through the use of mobile vans and containers, which are situated within central regions of the community. The study also noted the use of home HIV testing and counseling, which involves door-to-door testing and counseling services. The last one based on the study is multi-disease campaigns, which are mainly short health campaigns, which are mainly mobile, which are integrated with health promotion and screening for the other diseases.

A study by Mills *et al*. [2] on engaging men when it comes to prevention and care for HIV/AIDS in Africa noted that one of the strategies, which have been used is encouraging men to get tested and treated. However, this has not been done in a highly effective manner. The study further notes that despite the fact that various efforts have been put in place with the aim of understanding men's health-seeking behavior, they are still poorly understood when it comes to the AIDS epidemic. The study also notes that while there have been various approaches, there are generally no balanced approach when it comes to gender programming, and this has hindered effective involvement of both men and women in prevention of HIV and also in its treatment. The interventions, which have been used, have concentrated on women, due to the fact that women have been regarded to be predominantly vulnerable to HIV infection due to various biological factors, like their reduced sexual autonomy, as well as because of men's sexual power and privilege over them [3-7]. As a result, a number of the HIV/AIDS public health prevention and treatment campaigns have mainly focused on children and women. This has generally made men to received considerably minimal attention in the epidemic [8]. It has also made them to be less targeted when it comes to HIV prevention as well as to treatment program [6].

**Challenges faced. What has not worked?:** Sharma *et al*. argued that most of the interventions are always not tailored towards the needs of the men [1]. The scholars noted that for instance, a home HTC intervention within Botswana reached 85 per cent of women within the population, which was targeted in comparison to the 50 per cent of men [1]. The study noted that this was mainly brought about by the fact that the intervention took place during the workday and as a result, workingmen were missed. Neglect of men when it comes to the prevention and treatment campaigns of HIV has become one of the main challenges reported [2]. Different other studies have also indicated that men have been under-represented when it comes to HIV testing, HIV treatment, and HIV care. The studies have pointed out that this has various direct effects on the outcomes of care [9-13]. Despite the fact that there have been various public health efforts have mainly been aimed at women, and in particular on the childbearing women, scale-up efforts have been prevented by the differences in health-seeking behaviors between the men and women [14]. For example, sickness might be observed as a sign of weakness for a number of the men. This is generally a perception, which has made a number of men to be reluctant when it comes to care seeking among the men [15].

**What still needs to be done?:** various initiatives should be undertaken in order to improve the level of engagement of men in HIV services in sub-Saharan Africa. First, various measures should be adopted, which make sure that the health services, which are provided, are sensitive to the different needs of men. Secondly, various technologies, like mobile apps, social media, text messages and other kinds of campaigns ought to be used for engaging men. This ought to remind them concerning some of the main ways through which they are capable of protecting themselves and why they ought to engage in healthy behaviours. It can also encourage regular testing, as well as regular health check-ups.

**Figure 1:**
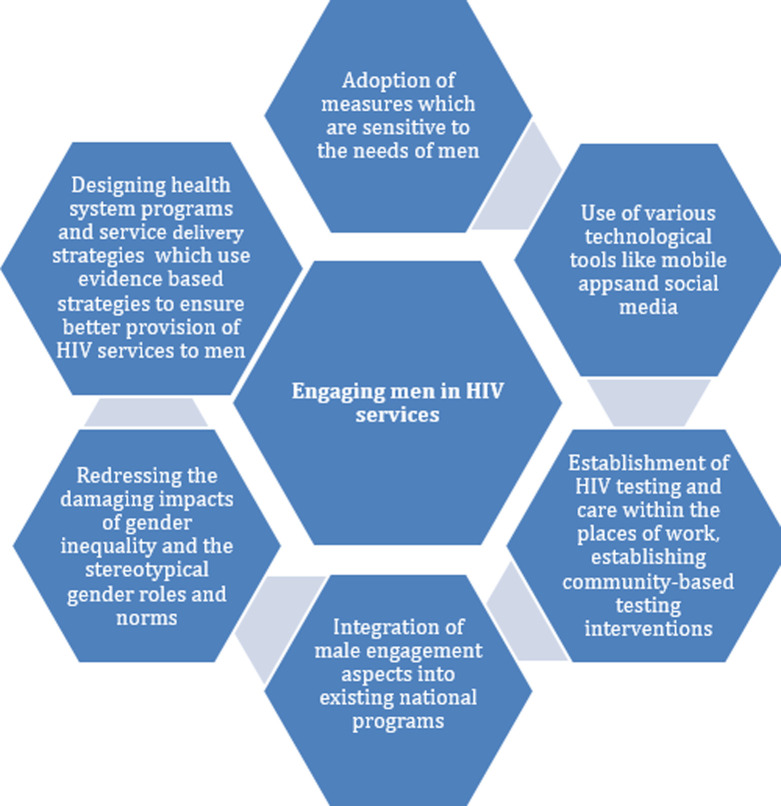
proposed building blocks to engagement of men in HIV services in sub-Saharan Africa

Funding agencies ought to recognize that women, as well as the males are both harshly impacted by the epidemic in different way. They ought to plan for various interventions, which engage both women and men. The relevant organizations, including the funding organizations ought to recognize the health and the social impacts, which are linked to lack of proper engagement of men in both primary and secondary HIV prevention campaigns. Programmatic efforts ought to take into consideration this disparity. Other innovative approaches that can be used for overcoming the challenges include establishment of HIV testing and care within the places of work and sports programs, ART home delivery, as well as HIV self-testing [16]. The other key interventions, which ought to be embraced generally, include integration of male engagement elements into the existing national programs, redressing the damaging impacts of gender inequality and stereotypical gender roles as well as the norms on the health and well-being of men. There is also the need to design health programs aimed at reaching the men. The men ought to be engaged all through their life cycles. The other notable initiative involves designing policies, as well as programs using evidence-based strategies and the available data. Figure 1 illustrates strategies proposed to engage men in HIV services in sub-Saharan Africa.
